# Supplementary figures and images for: Accumulation mechanisms of radiocaesium within lichen thallus tissues determined by means of in situ microscale localisation observation
Source: PLoS One. 2022 Jul 8;17(7):e0271035. doi: 10.1371/journal.pone.0271035 (PMC9269901; doi:10.1371/journal.pone.0271035)

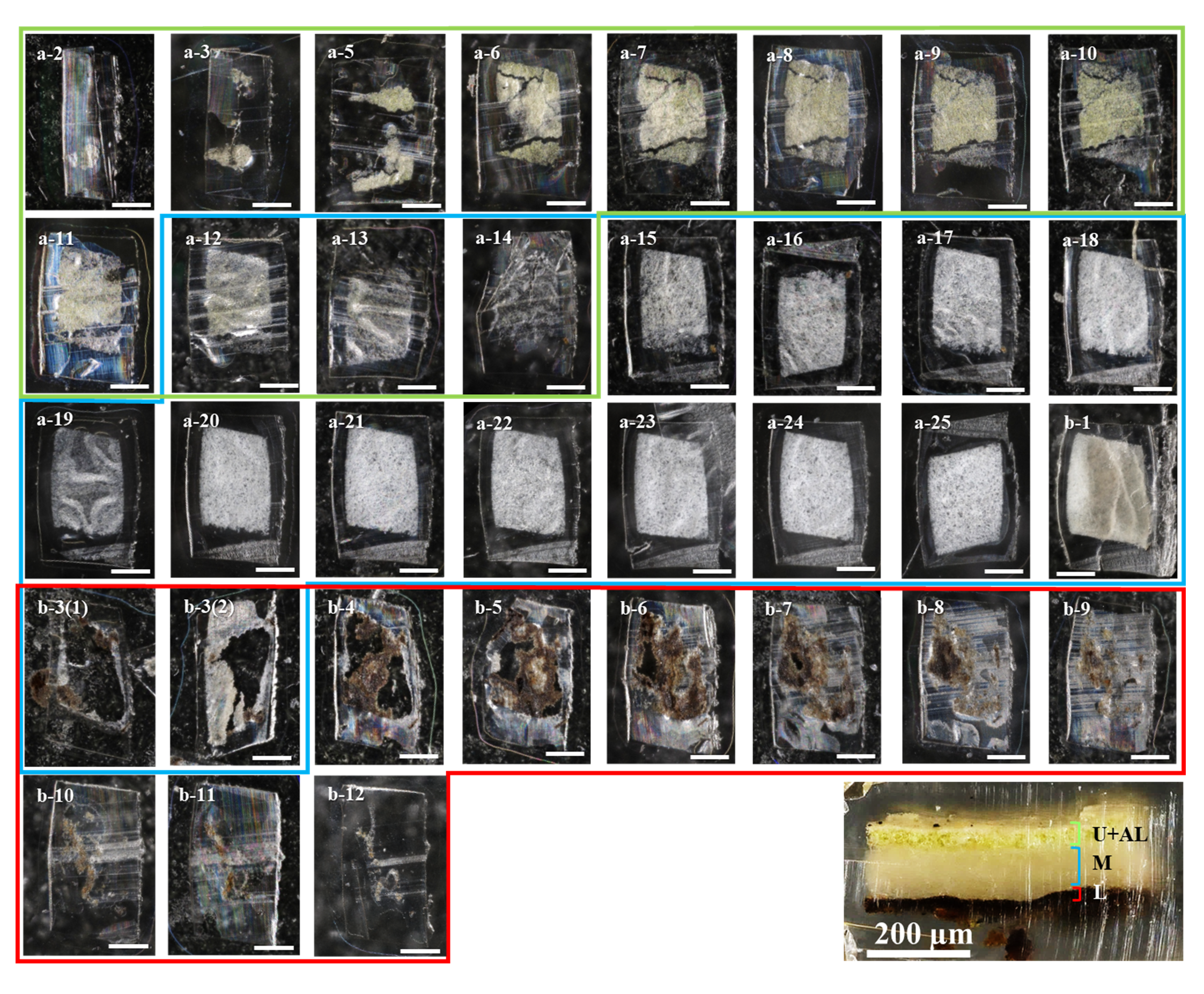

Supplement: S1 Fig — The tissue sections were prepared from the broad distribution “a” of the FY2017PT sample in Figs 1 and 2. The lower right panel image shows cross-sectional image same as Fig 5A. Autoradiograph images of these sections were shown in Figs 5 and 6, and S8–S12 Figs. Coloured frames are sections containing tissue. Green, aqua and red boxes indicate the areas corresponding to the upper cortex including the algal layer (U+AL in cross-sectional image), medullary layer (M) and lower cortex (L), respectively. Overlapping boxes indicate that two layers were contained in the frames. The “a-4” and “b-2” are missing numbers. The “b-3” layer contains 2 sections. Bar = 200 μm. (TIF) [file pone.0271035.s001.tif]

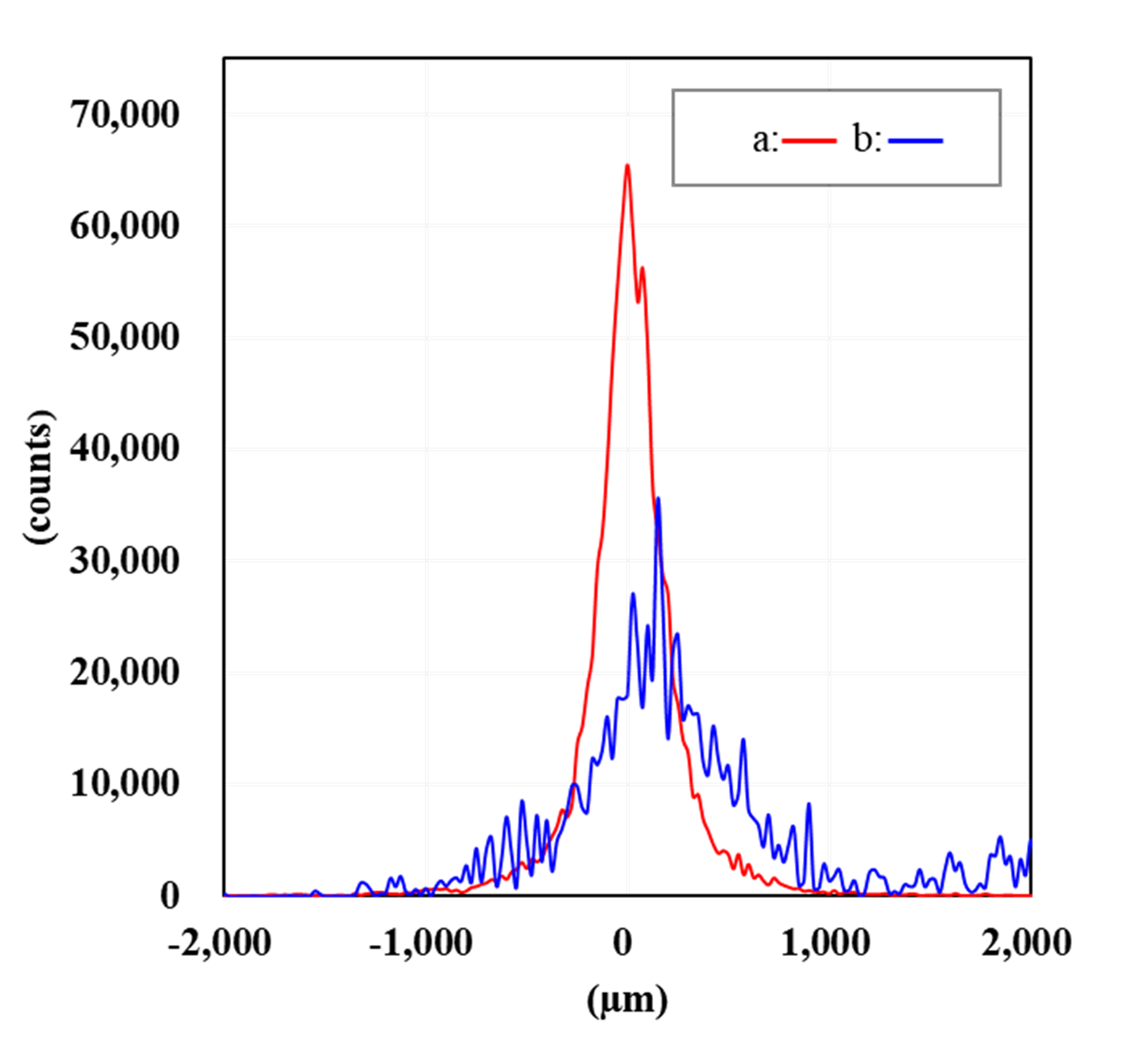

Supplement: S2 Fig — The red line “a” shows the pattern of the CsMP trapped in lichen (FY2016CsMP), which was previously determined by Dohi et al. [21]. The CsMP sample was exposed for the 16 hours on the IP. The blue line “b” shows the pattern of the Cs-containing particle in Fig 12. The linear intensity through the centre (x-axis aligned with 0) of each autoradiogram spot is shown as an example. The x-axis indicates linear distance of the distribution (μm), and the y-axis indicates the PSL signal intensity values (counts) in both graphs. (TIF) [file pone.0271035.s002.tif]

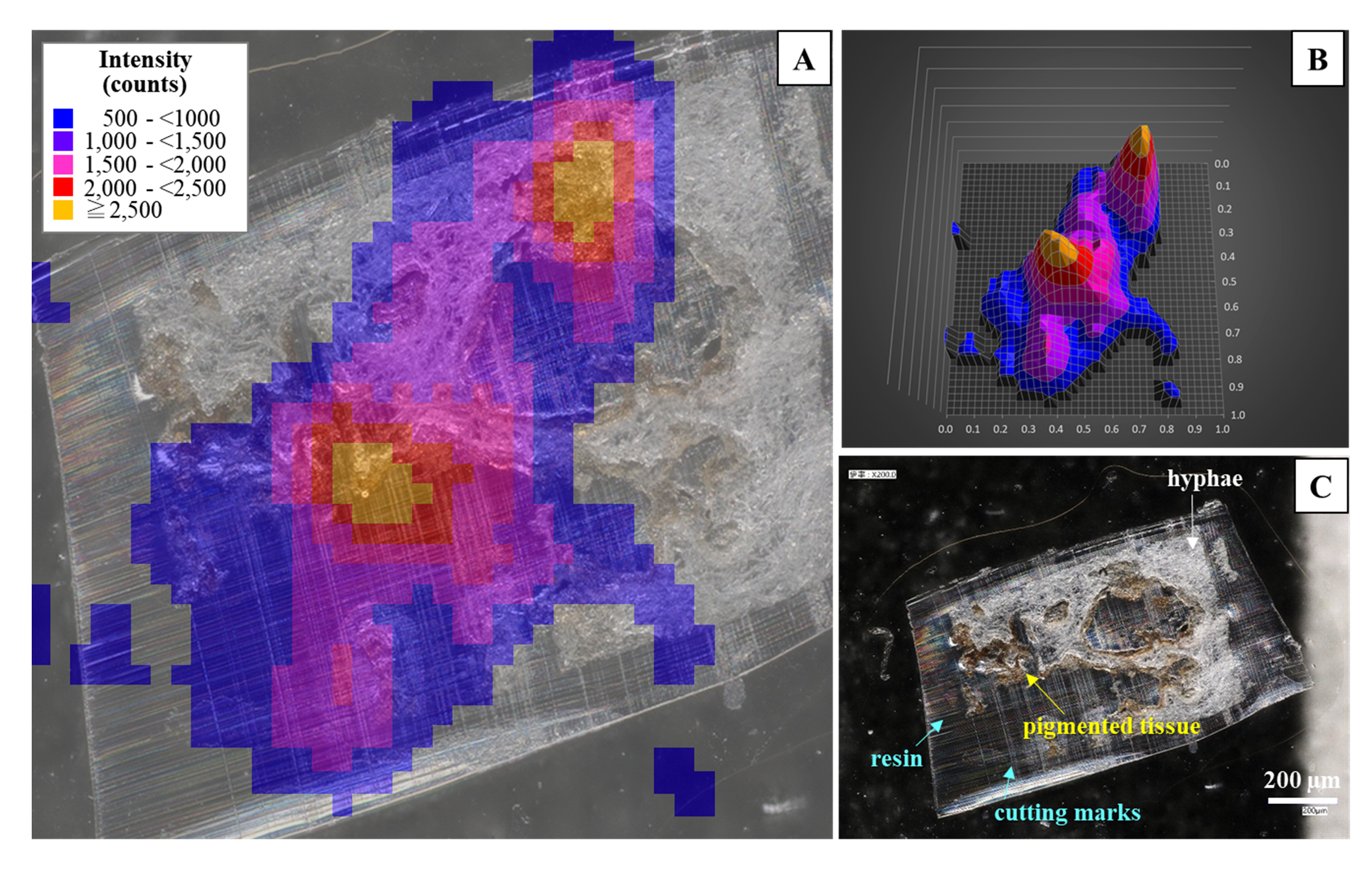

Supplement: S3 Fig — The radiocaesium distributions of the “c-3” to “c-8” layers in the FY2012PT sample. The “c-4” layer was excluded here (see Fig 4). S3–S7 Figs show c-3 to c-8 layers, respectively. (A) Overlapping image of the autoradiography result and tissue section image. Background counts were deducted from all count values. Each colour indicates the range of the PSL signal intensities, as shown in the legend, which also applies to panel (B). (B) Three-dimensional image of (A) in a 1 mm square area. (C) Image of tissue section taken with the digital microscope. The brown parts are pigmented tissue (yellow arrow), white parts are hyphae (white arrow), and transparent parts are resin (aqua arrow). Hereinafter the same within S3–S7 Figs. Cutting marks from the glass knife appeared as a white linear pattern on the resin. (TIF) [file pone.0271035.s003.tif]

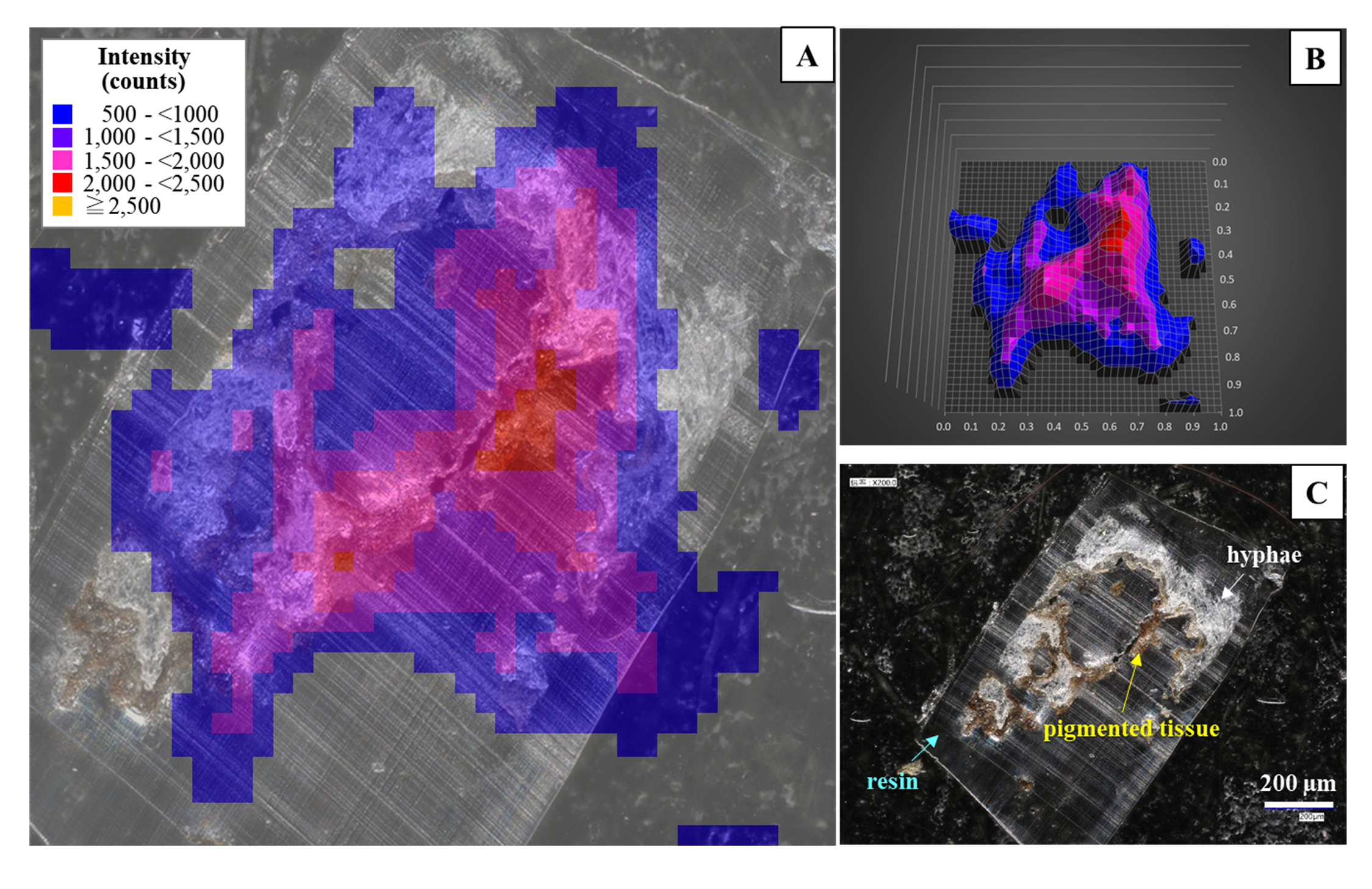

Supplement: S4 Fig — (TIF) [file pone.0271035.s004.tif]

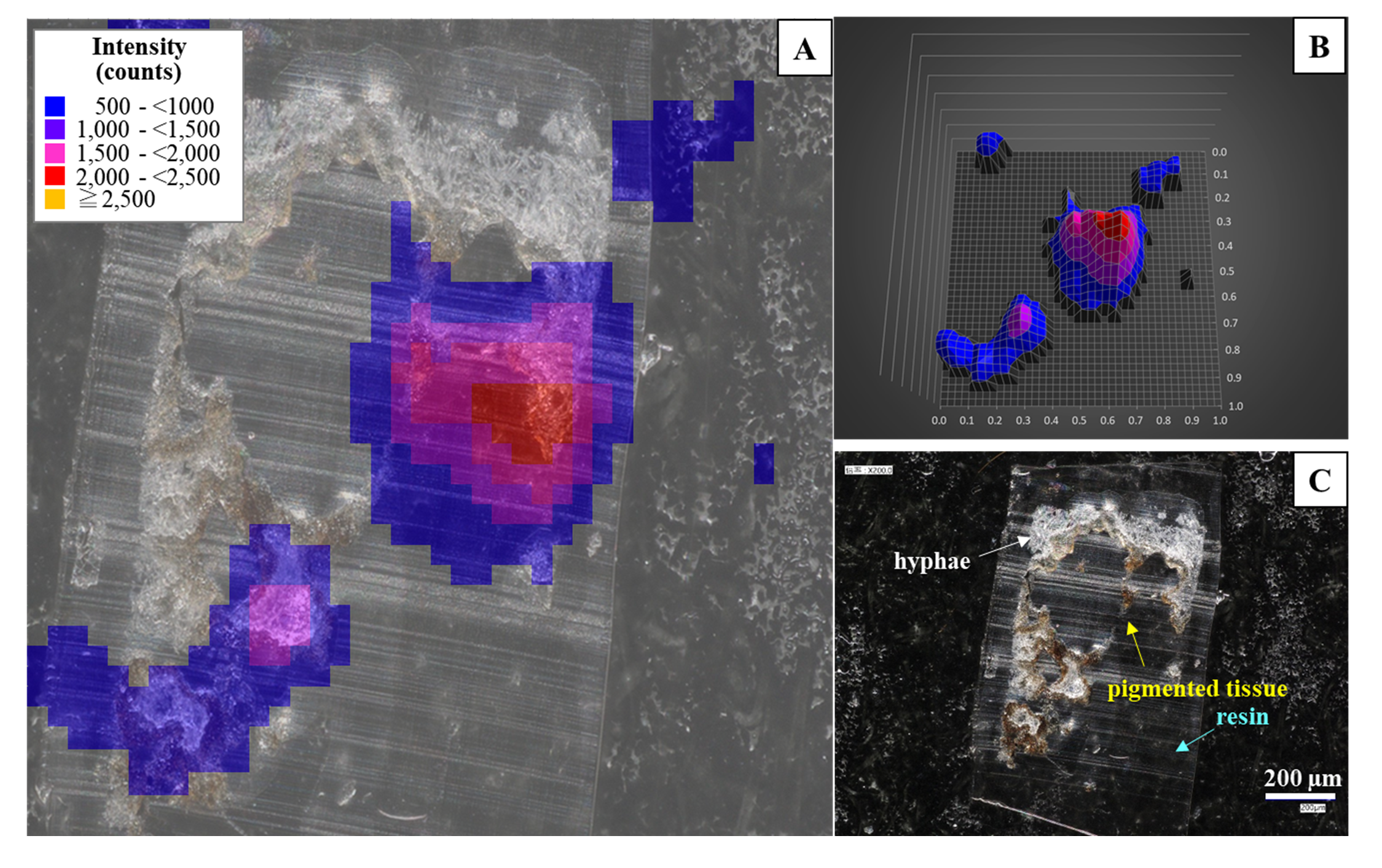

Supplement: S5 Fig — (TIF) [file pone.0271035.s005.tif]

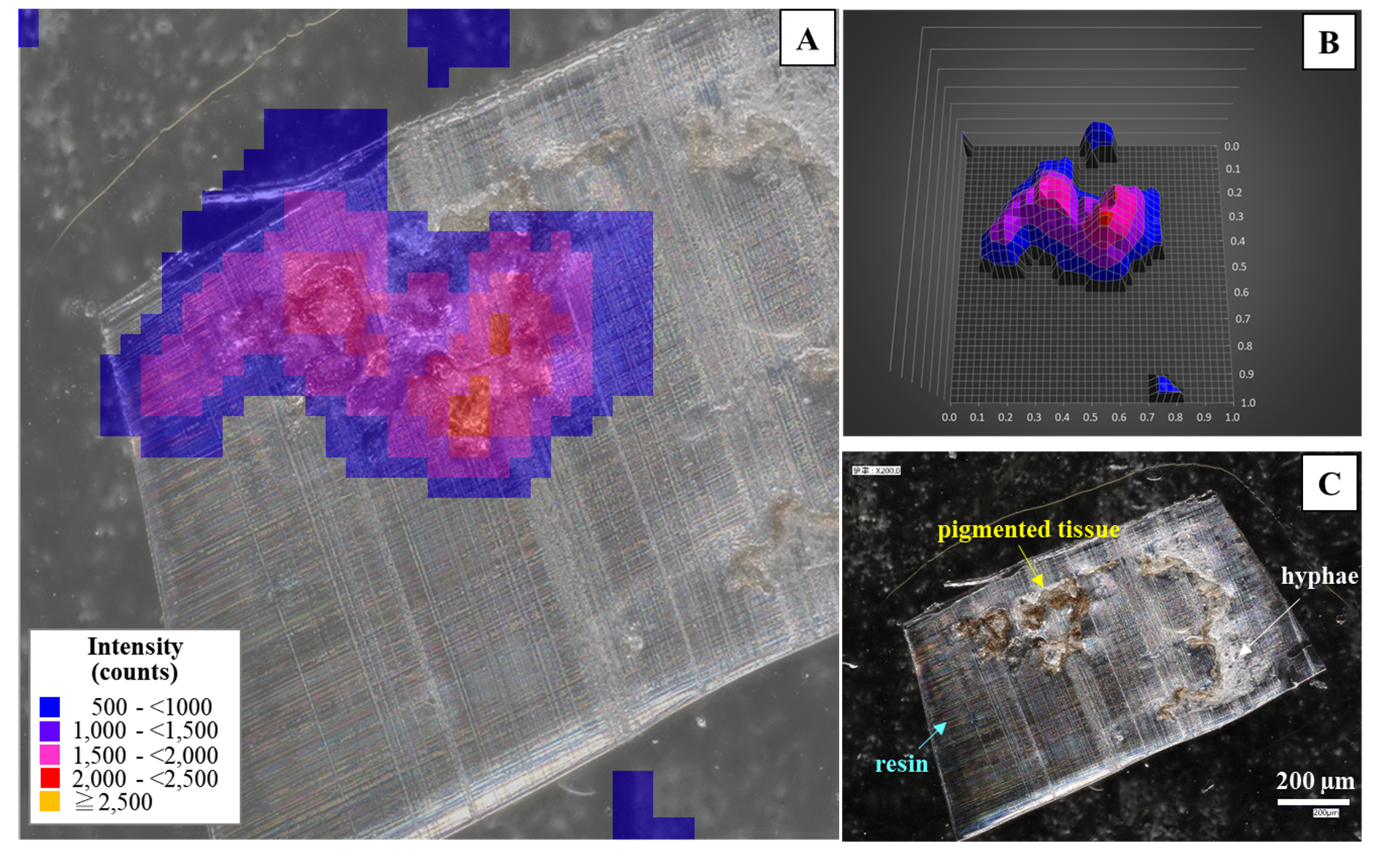

Supplement: S6 Fig — (TIF) [file pone.0271035.s006.tif]

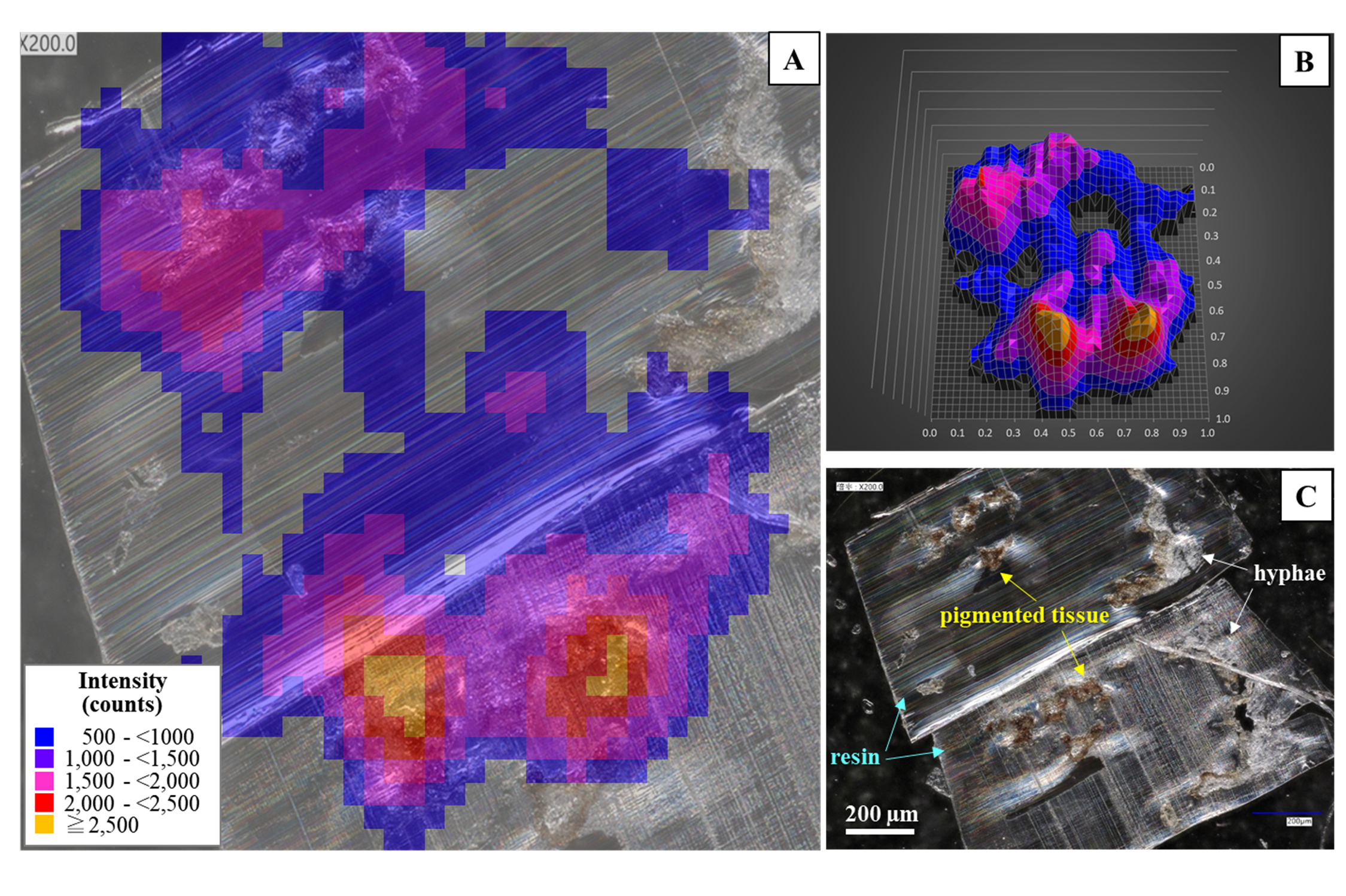

Supplement: S7 Fig — (TIF) [file pone.0271035.s007.tif]

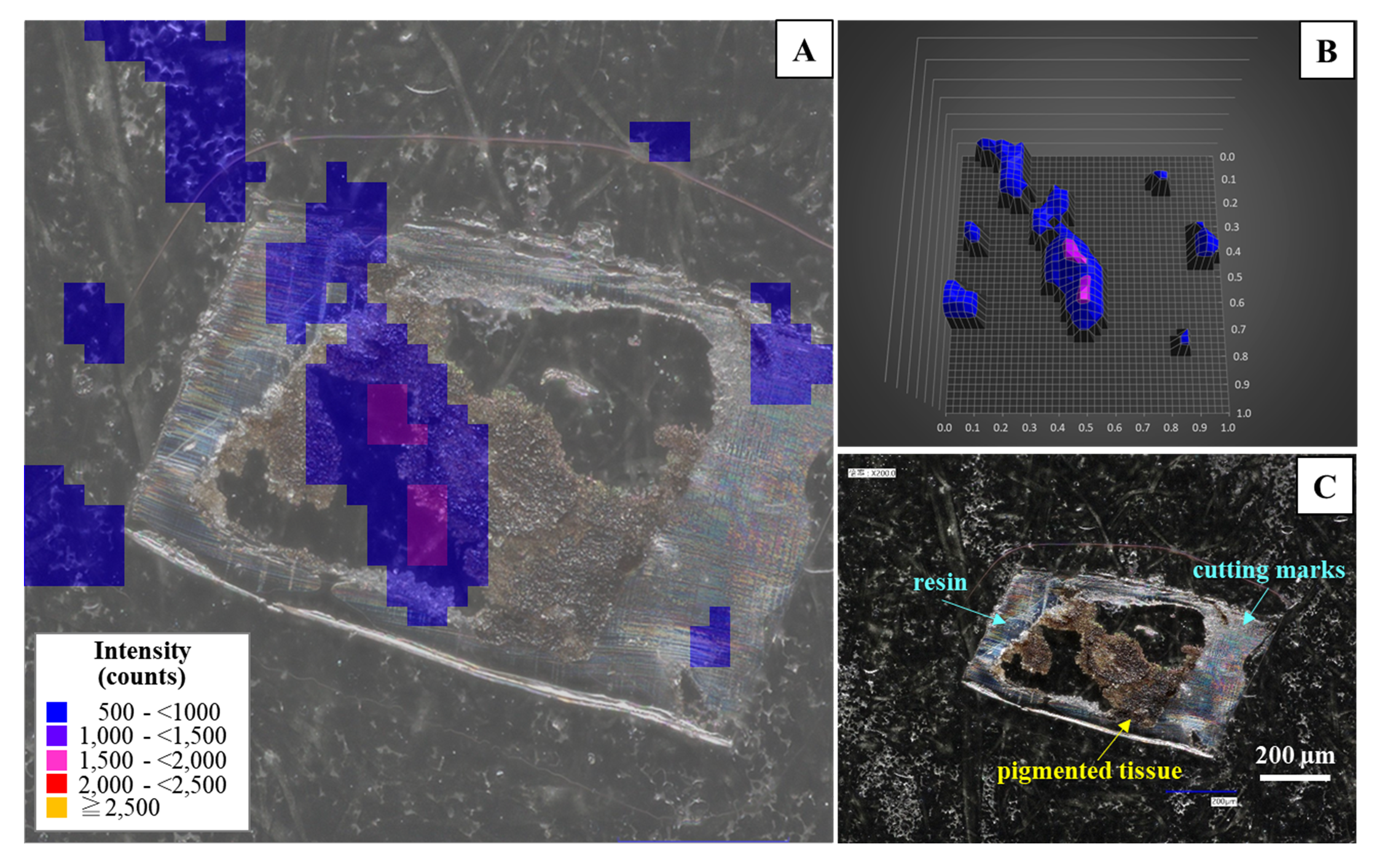

Supplement: S8 Fig — The radiocaesium distributions of the “b-4” to “b-9” layers in the FY2017PT sample. The “b-5” layer was excluded here (see Fig 6). S8–S12 Figs show b-4 to b-9 layers, respectively. (A) Overlapping image of the autoradiography result and tissue section image. The background counts were deducted from all count values. Each colour indicates range of the PSL signal intensities, as shown in the legend, which also applies to panel (B). (B) Three-dimensional image of (A) in a 1 mm square area. (C) Image of tissue section taken with the digital microscope. The brown parts are pigmented tissue (yellow arrow), and transparent parts are resin (aqua arrow). Hereinafter the same within S8–S12 Figs. Cutting marks from the glass knife appeared as a white linear pattern on the resin. (TIF) [file pone.0271035.s008.tif]

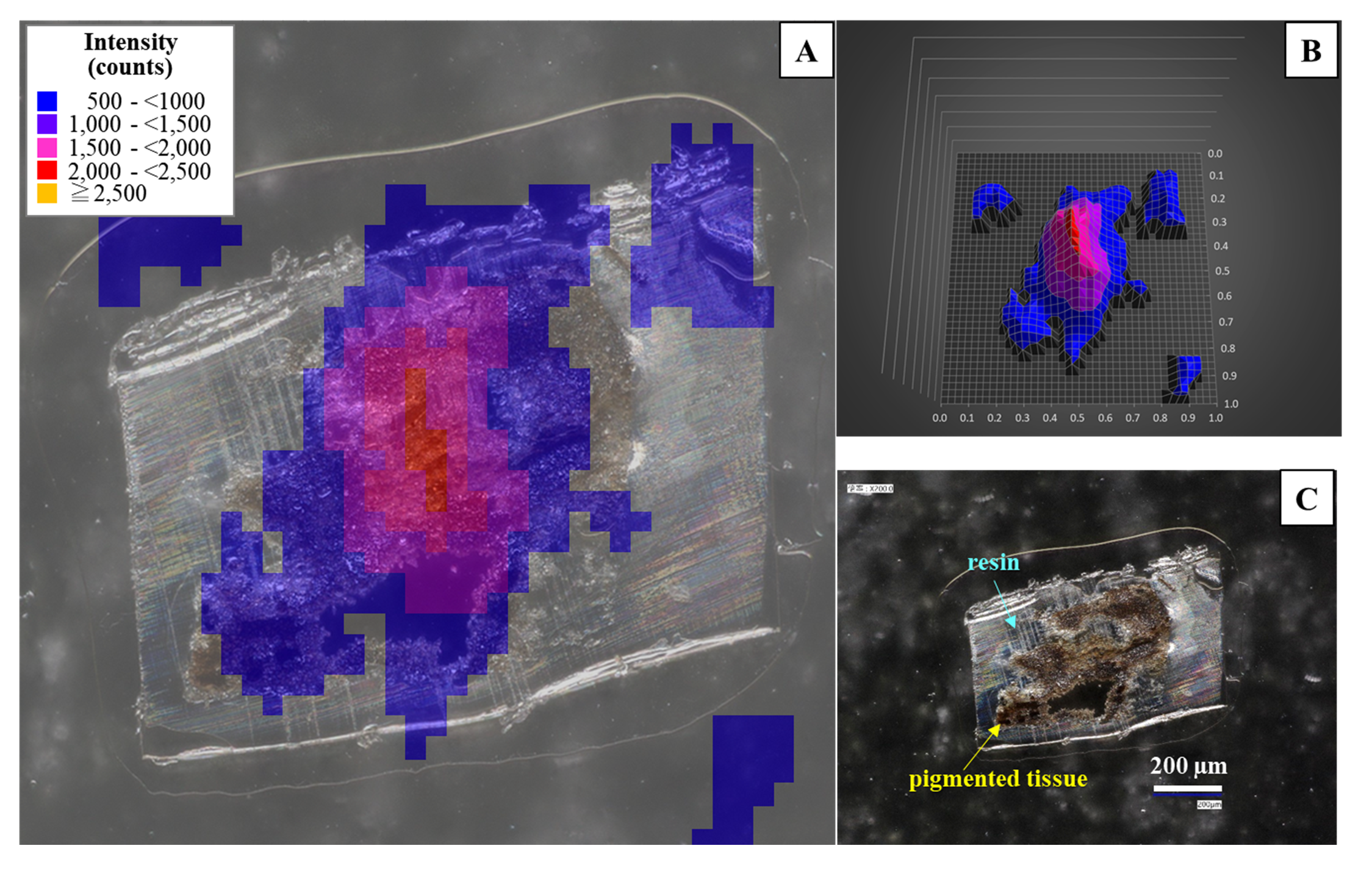

Supplement: S9 Fig — (TIF) [file pone.0271035.s009.tif]

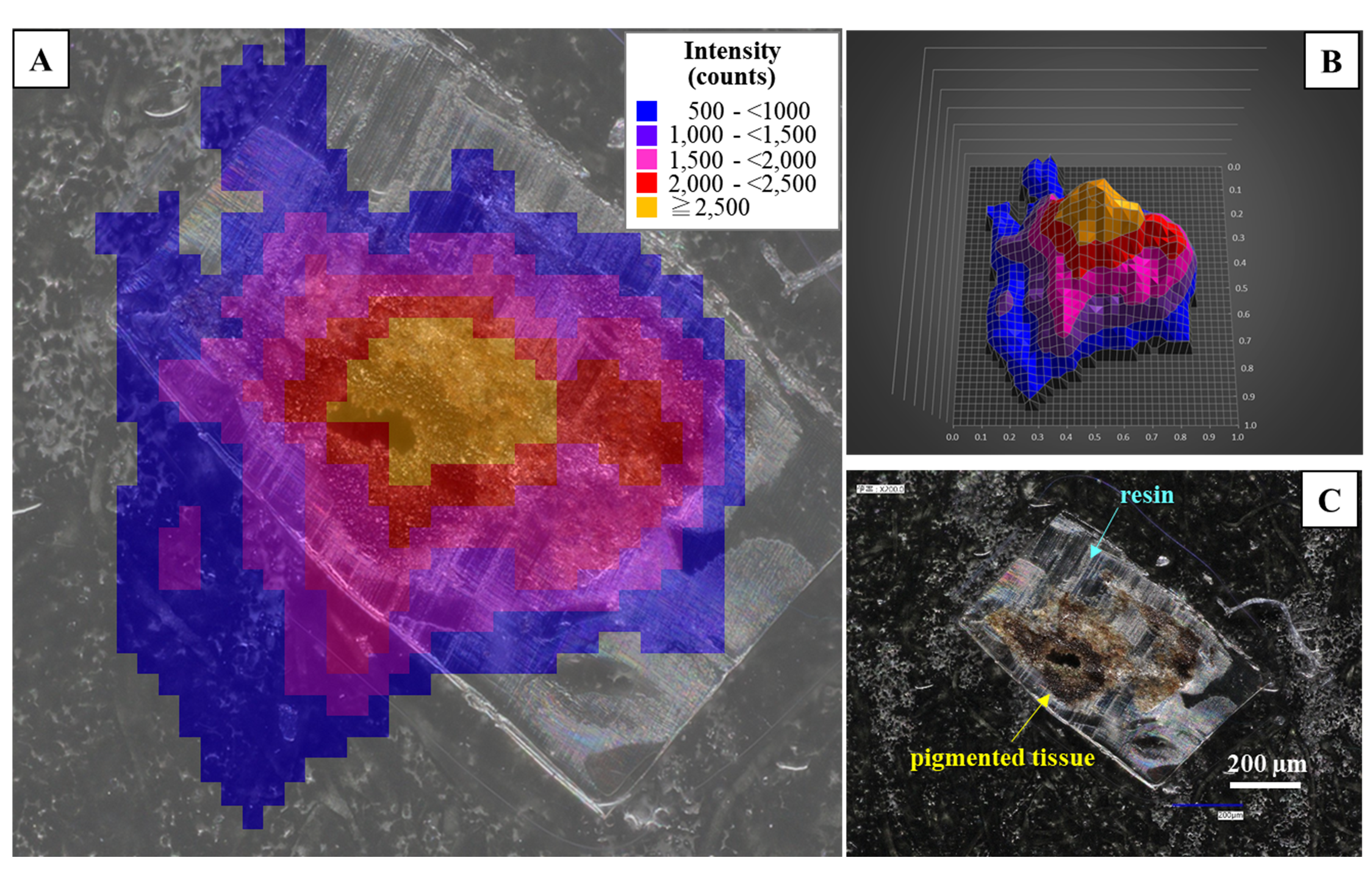

Supplement: S10 Fig — (TIF) [file pone.0271035.s010.tif]

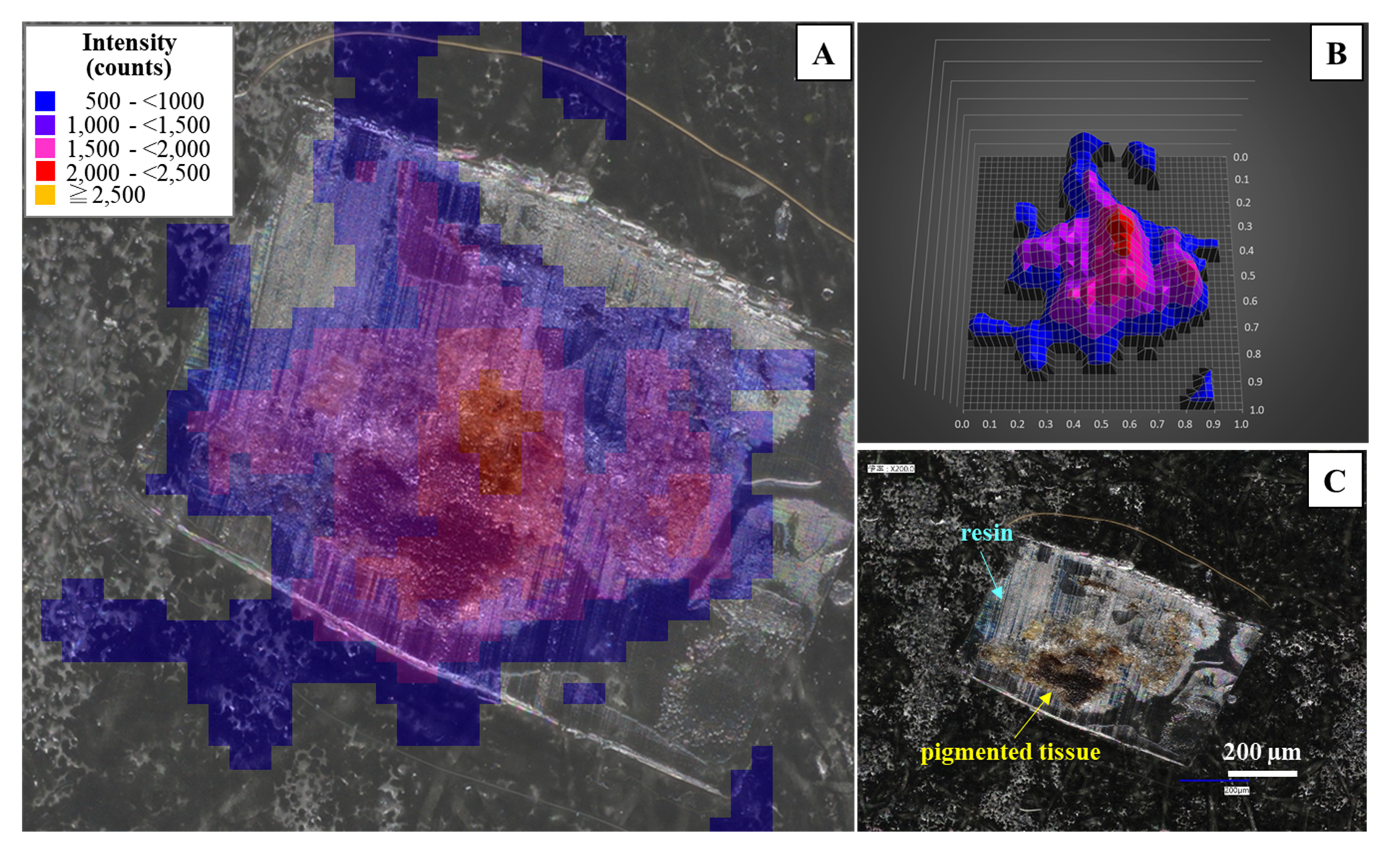

Supplement: S11 Fig — (TIF) [file pone.0271035.s011.tif]

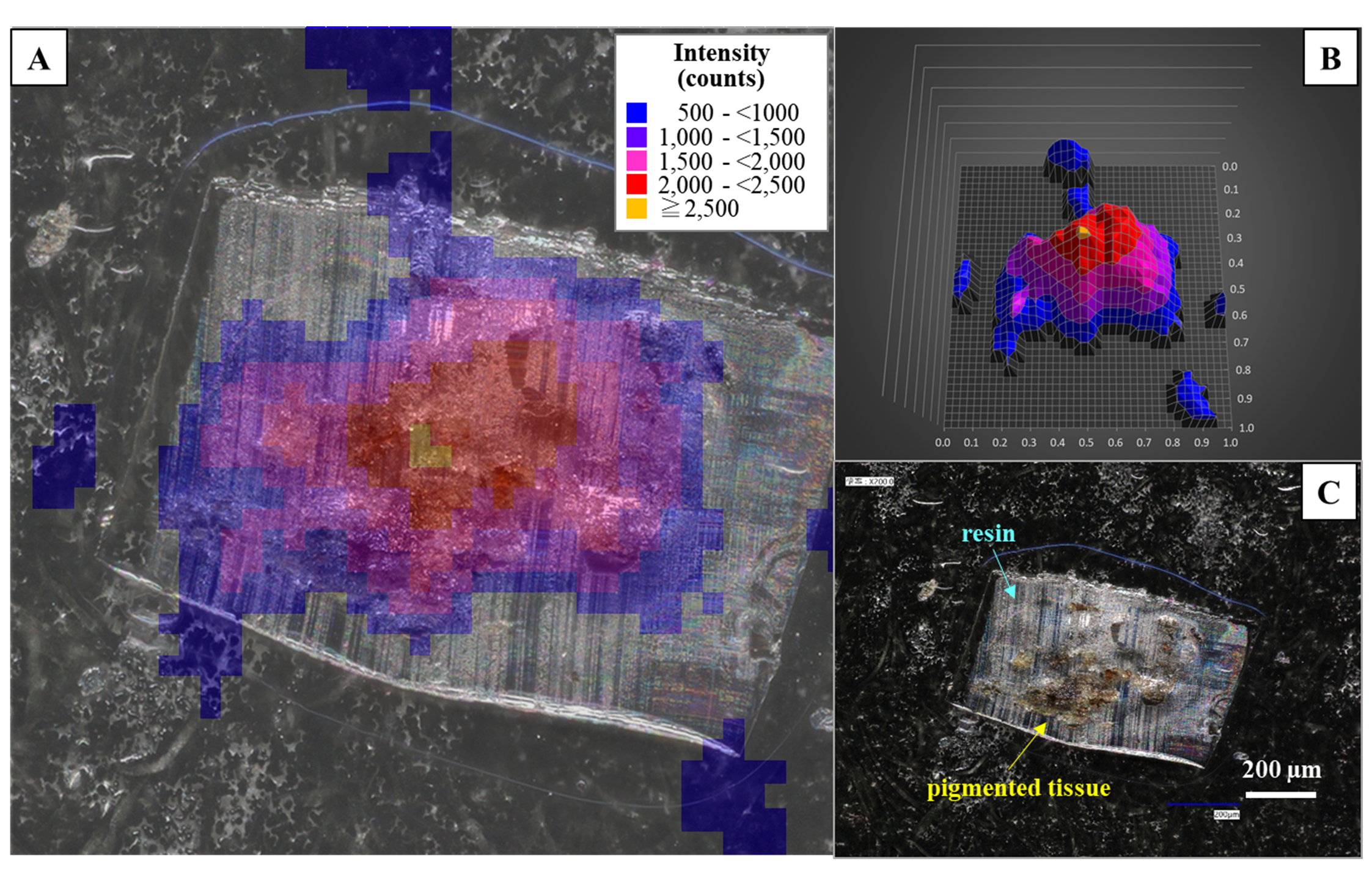

Supplement: S12 Fig — (TIF) [file pone.0271035.s012.tif]

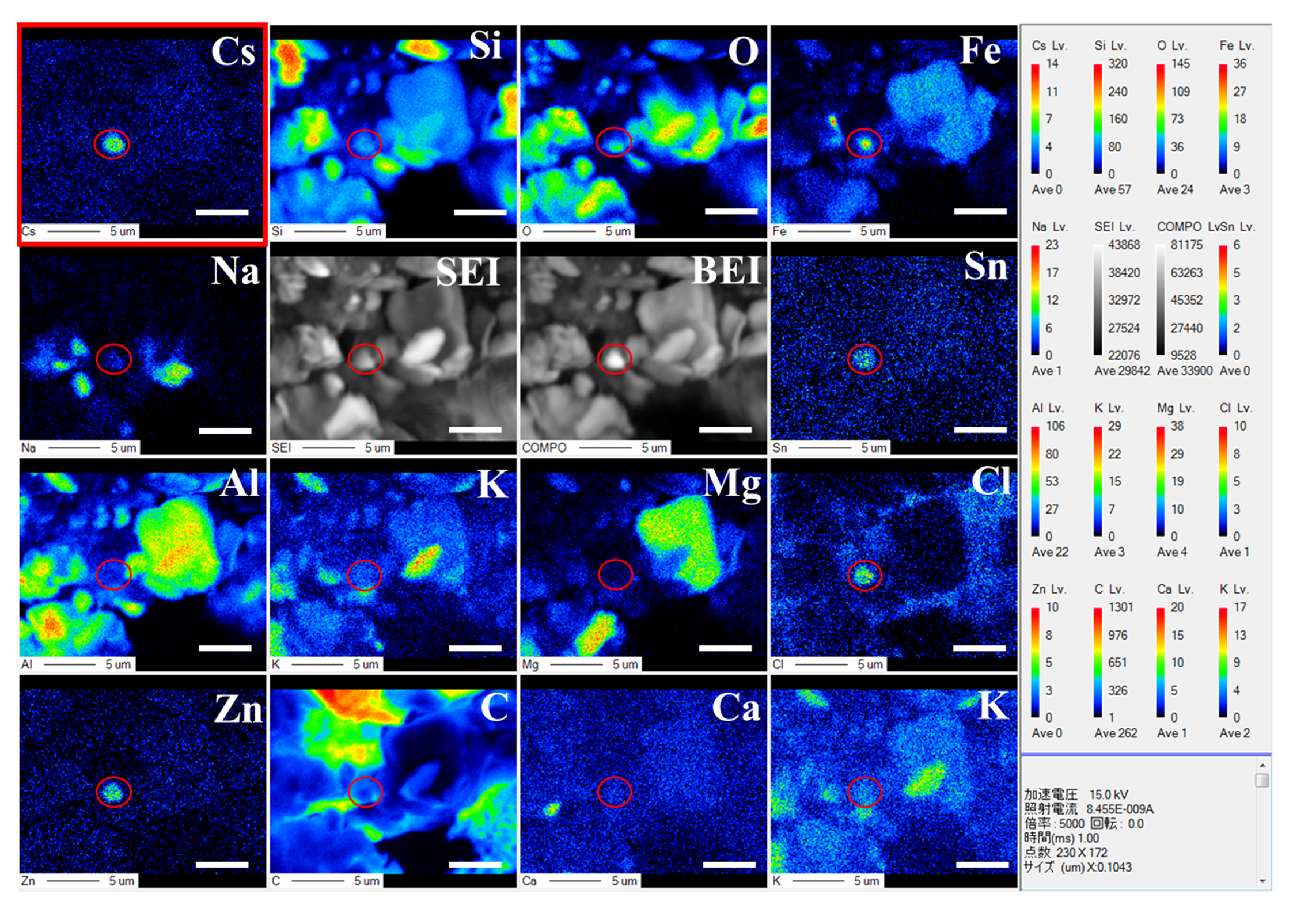

Supplement: S13 Fig — This analysis was carried out to determine a Cs-containing particle (red circle) and its co-existing elements on the thallus surface of the FY2012PT sample using FE-EPMA-WDS. (TIF) [file pone.0271035.s013.tif]
