# Supplementary material for: Accumulation mechanisms of radiocaesium within lichen thallus tissues determined by means of in situ microscale localisation observation
Source: PLoS One. 2022 Jul 8;17(7):e0271035. doi: 10.1371/journal.pone.0271035 (PMC9269901; doi:10.1371/journal.pone.0271035)
Supplement: Attachment — Submitted filename: Response 2 to Reviewers_20220616_Dohi et al.pdf [file pone.0271035.s014.pdf]

Question from Reviewer #2 related to L234-236

The radiocaesium (134, 137Cs) activities were determined based on standard point sources, CZ402 for 134Cs and CS402 for 137Cs (Japan radioisotope association, Tokyo, Japan) [21]. The standard point sources (CZ402 and CS402) have 6mm diameter. On the other hands, CsMPs have a few μm diameter. Do the standard point sources have enough accuracy to determine the radioactivity of CsMPs?

Answer

Regarding to the geometric efficiency, there is no significant difference between the standard point sources and CsMP.

Matsui *et al.* (1973) reported the ratio of the geometric efficiency of disc sources, G_s, to that of point source, G_0, with various diameter of sample, R, diameter of detector, A, and height of sample position, Z [r1].

In our measurement system, each standard point source has 6 mm diameter and 5 mm height from the bottom against 76 mm diameter of the detector.
The samples were set by the modified polypropylene holder so as to be fixed also at 5 mm height from the center of the detector surface.
These conditions give fixed value of Z/A=0.13 for both standard point source and CsMP sample, and R/Z=0.6 for standard point source and 0.0002 for CsMP sample.

According to Fig.9 of Matsui et al. (1973), the geometric efficiency ratio, G_s/G_0, in case of CsMP (R/Z=0.0002) was almost equal to 1, while, that of standard disc source (R/Z=0.6, Z/A=0.13) was also likely to 1, since it was considered to be larger than that of R/Z=0.6, Z/A=0.5, which was estimated around 0.98 based on the figure.
It indicates that there is no significant difference of geometric efficiency between the standard point sources and CsMP.

Reference

[r1] Matsui Y and Furuta T., Geometrical detection efficiency of point and disc source. RADIOISOTOPES (in Japanese). 1973; 22: 12: 682-689.

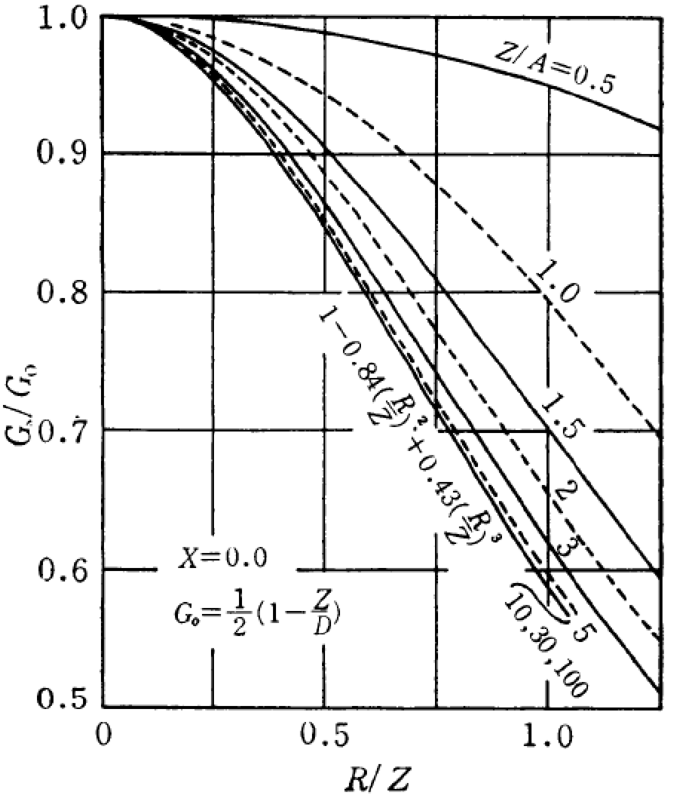

Fig. 9 Ratio of geometrical efficiency of a disc source to that of a point source (on the center line).
